# Myricetin Improves Impaired Nerve Functions in Experimental Diabetic Rats

**DOI:** 10.3389/fendo.2022.915603

**Published:** 2022-07-19

**Authors:** Junxiong Ma, Jun Liu, Yu Chen, Hailong Yu, Liangbi Xiang

**Affiliations:** Department of Orthopedics, General Hospital of Northern Theater Command, Shenyang, China

**Keywords:** myricetin, DPN, nociception, oxidative stress, Nrf2

## Abstract

Diabetic peripheral neuropathy (DPN) is considered as one of the most important complications of diabetes mellitus. At present, effective treatments that might improve the damaged neurological function in DPN are sorely needed. As myricetin has been proved to possess excellent neuroprotective and antioxidant effects, it might have therapeutic potential for DPN. Therefore, the purpose of our study was to detect the potential beneficial effect of myricetin on DPN. A single dose of 50 mg/kg of streptozotocin was applied in rats for the establishment of diabetic models. Different doses of myricetin (0.5 mg/kg/day, 1.0 mg/kg/day, and 2.0 mg/kg/day) were intraperitoneally injected for 2 weeks from the 21st day after streptozotocin injection. After the final myricetin injection, behavioral, electrophysiological, biochemical, and protein analyses were performed. In the present study, myricetin significantly ameliorated diabetes-induced impairment in sensation, nerve conduction velocities, and nerve blood flow. In addition, myricetin significantly reduced the generation of advanced glycation end-products (AGEs) and reactive oxygen species (ROS), and elevated Na^+^, K^+^-ATPase activity and antioxidant activities in nerves in diabetic animals. Additional studies revealed that myricetin significantly raised the hydrogen sulfide (H_2_S) levels, and elevated the expression level of heme oxygenase-1 (HO-1) as well as nuclear factor-E2-related factor-2 (Nrf2) in diabetic rats. In addition, myricetin has the capability of decreasing plasma glucose under diabetic conditions. The findings in our present study collectively indicated that myricetin could restore the impaired motor and sensory functions under diabetic conditions. The Nrf2-dependent antioxidant action and the capability of decreasing plasma glucose might be the underlying mechanisms for the beneficial effect of myricetin on impaired neural functions. Our study showed the therapeutic potential of myricetin in the management of DPN.

## Introduction

As one of the most common and important global health effects, diabetes mellitus (DM) affects more than 366 million people worldwide (1). Diabetic peripheral neuropathy (DPN) is considered as one of the most important complications of DM, affecting more than half of diabetic patients. DPN is characterized by severe hyperalgesia, allodynia, and impaired sensory and motor nerve conduction velocities (NCV), which might be painful, disabling, and even fatal (2). Analgesics could be used for nociceptive pain, but common analgesics are often partly effective in reducing nociceptive pain, and hardly exhibit any beneficial effect on impaired motor NCV in the majority of patients. Thus far, no effective treatment for DPN is available besides a strict control of glycemia. Therefore, effective medicines for painful DPN are sorely needed.

Oxidative damage is the most important and most common pathway in the pathophysiological processes of diabetes and related complications (3). Excessive production of reactive oxygen species (ROS) was proved to be a capital component during the whole processes of DPN (3, 4). Therefore, antioxidants that are capable of attenuating oxidative damage to axons and neurons hold the potential in alleviating diabetic neuropathy. Recently, a large number of evidence indicates that the more targeted the approach, the more optimized neuroprotection it provides in DPN. The approach targeting nuclear factor-E2-related factor-2 (Nrf2), which is thought to be the axis of antioxidative defense, has been one of the focus of DPN (5). Thus far, Nrf2 has been shown to be involved in the protection of peripheral nerve (6), pancreatic β cells (7), kidney (8), and cardiomyocytes (9) against oxidative damage under diabetic conditions. Therefore, antioxidants that could regulate transcriptional activity of Nrf2 to inhibit oxidative damage may provide a more promising avenue in DPN.

Myricetin (3,5,7,3’,4’,5’-hexahydroxyflavone cannabiscetin) exists widely in nature, such as vegetables, fruits, berries, tea, medical plants, and red wine. As a kind of natural flavonol, the daily intake of myricetin (1.0 mg/day) is higher than others (10). The unique structure of hydroxyl groups at 3,5 positions as well as 3 consecutive hydroxyl groups at 3’,4’,5’ increases its antioxidant effectiveness. It has been proved that myricetin could scavenge ROS in both *in vitro* and *in vivo* studies (10, 11). In addition, myricetin has also been shown to protect kidney and pancreatic β cells against oxidative damage under diabetic conditions (11, 12). A microarray and pathway analysis has shown that myricetin is capable of activating the Nrf2 pathway in hepatic cells (13). All those findings suggest that myricetin holds the potential to manipulate the Nrf2 pathway to counteract oxidative damage. In addition, myricetin has been proved to possess neural protective effects in neurons in recent years (14). Taken together, the Nrf2-mediated antioxidant ability and neuroprotective characteristics of myricetin suggest its clinical applicability to DPN. Therefore, the purpose of our present study was to detect the potential beneficial effects of myricetin on DPN in a rat model.

## Experimental Procedures

### Animal Model of DPN

All animal experiments were performed in line with the guidelines of the China Council on Animal Care and Use. This study obtained approval from the Committee of Experimental Animals of General Hospital of Northern Theater Command (approval number: GK20190920) on September 20, 2019. Adult male Sprague–Dawley rats (200 g to 240 g, Experimental Animal Center, General Hospital of Northern Theater Command, Shenyang) were used in the present research. A painful DPN model (*n* = 40) was established by intraperitoneal streptozotocin (STZ) injection (50 mg/kg). The same volume/kg saline was intraperitoneally injected in the normal control group (*n* = 10). From the 21st day after STZ injection, blood glucose levels were measured before noon twice a week from tail vein samples using a commercially available glucometer (Freestyle, Abbott Diabetes Care, Inc.; Alameda, CA, USA). Plasma glucose was measured to confirm the establishment of the diabatic model, which was judged according to the diagnostic criteria of glucose of more than 13.89 mmol/L. Furthermore, DPN with allodynia and hyperalgesia was confirmed in the STZ group at 21 days after STZ treatment. The diabatic rats were then randomly divided into an STZ control group, a low-dose myricetin group (0.5 mg/kg/day), a mid-dose myricetin group (1.0 mg/kg/day), and a high-dose myricetin group (2.0 mg/kg/day) (*n* = 10 for each group). For the myricetin group, myricetin (2 ml of saline solution) was daily administrated for 2 weeks. For the STZ control group and normal group, 2 ml of saline without myricetin was daily administrated for 2 weeks. After the final myricetin administration, behavioral analysis, electrophysiological examination, and biochemical and protein analysis were achieved. After all analyses, plasma glucose was examined through ELISA (Sigma-Aldrich, St. Louis, MO, USA). Streptozotocin and myricetin were purchased from Sigma Chemical Co., St. Louis, MO, USA.

### Mechanical Nociception

The mechanical sensation was examined before STZ administration as baseline test. The test was repeated every 3 days after STZ administration and on the 13th day after myricetin administration. The von Frey filament was used to examine the mechanical sensitivity (15). A special cage with a wire-mesh bottom was prepared. In brief, after placing the rats in the cage, a 0.5-mm von Frey filament was used to evoke nociceptive responses by sticking the plantar aspect of the foot pads of the hind paw. The filament was applied for 3 to 5 s, which was repeated 10 times with 30 s of inter-stimulus interval (**Supplementary Figures 1 A, B**). Be careful to avoid continuous stimulation of the same location of the foot pads. The positive response was defined as abrupt withdrawal and licking of the paws. The number of positive responses out of 10 stimuli was recorded at 0, 1, 2, 3, 4, 5, and 6 h after the final myricetin injection. If the ratio of positive responses increased significantly, the rats were considered to suffer from mechanical hyperalgesia.

### Heat Nociception

The heat nociception was assessed according to latency to heat stimuli (16) before STZ administration as baseline test. The test was repeated every 3 days after STZ administration and on the 14th day after myricetin administration. In brief, the nociceptive response to heat stimulation was tested through a thermal stimulation system (**Supplementary Figures 1C, D**). The plantar aspect of the foot pads of the hind paw was stimulated by a radiant heat source (46 to 48°C). The latency to heat stimulation was recorded as the paw withdrawal latency (s). When the rats did not withdraw the paw until 20 s, the test was stopped in order to avoid heat injury. The stimulation was repeated 3 times with 5 min of inter-stimulus interval. The latency to heat stimulation was recorded at 0, 1, 2, 3, 4, 5, and 6 h after the final myricetin injection.

### Cold Allodynia

The cold allodynia was assessed according to latency to cold stimuli before STZ administration as baseline test. The test was repeated every 3 days after STZ administration and on the 15th day after myricetin administration. In brief, a homeothermic stainless steel plate (10°C, model 35100, Ugo Basile) was prepared for the cold stimulation. The latency to cold stimuli is recorded as paw withdrawal latency (s). Paw withdrawal was defined as lifting or shaking of the hind paws when the rats were placed on the plate. When the rats do not withdraw their paw for 30 s, the test was stopped in order to avoid congelation. The stimulation was repeated twice with 5 min of inter-stimulus interval. The latency to cold stimulation was recorded at 0, 1, 2, 3, 4, 5, and 6 h after the final myricetin injection.

### Nerve Blood Flow

When all the behavioral tests above were finished, the right sciatic nerve of the rat was exposed under general anesthesia (pentobarbital, 40 mg/kg). Then, a laser Doppler flowmeter (Hpsonos 1500, USA) was used to detect the NBF with a laser probe that was laid on the nerve. When recording the NBF, the temperature of the animals was kept at 37°C (17).

### Sciatic NCV

Thirty minutes after the NBF test, the motor NCV and sensory NCV were measured sequentially. First, a stimulating electrode and recording electrode were put under the nerve with 10 mm of interval between the two electrodes. By using a single pulse (1.2 V, 1.0 ms) stimulating the nerve, the motor NCV was calculated through the PowerLab 4SP distal data acquisition system (Keypoint 3.02 Denmark). Then, the position of the stimulating electrode and recording electrode was switched. The sensory NCV was then calculated in the same way as above (17). To investigate whether NBF measurement has an influence on NCV, the NCV measured from rats that had undergone NBF was compared to that measured without the NBF procedure. No significant difference was found between the values of NCV with or without NBF procedures (**Supplementary Figure 2**).

### Histology

After electrophysiological studies, the sciatic nerve was exposed and fixed in 4% glutaraldehyde for 5 min. The nerve was post-fixed in osmium tetroxide (1%) in pH 7.3 sodium cacodylate buffer (0.1 M). After being dehydrated in ethanol, the samples were embedded in resin. One-micrometer-thick semi-thin sections and 50-nm-thick ultra-thin sections were produced from the distal part of the sample. A light microscope (AH3, Olympus, Tokyo, Japan) was used to examine the toluidine blue/borax-stained semi-thin sections. The H-600 transmission electron microscope (HITACHI, Tokyo, Japan) was employed to examine the ultra-thin sections stained by uranyl acetate/lead citrate. Morphometric evaluations were conducted by examiners who were blinded to the experimental design. For each sample, at least 3 random high-power fields were selected for observation and calculation. Axonal myelination was estimated by the area of the separation between myelin and axoplasm through ImageJ software (Media Cybernetics, Rockville, MD). If the area of the separation between myelin and axoplasm was more than 6% of the cross section of the axon, it is defined as “axon–myelin separation” (18).

### Na^+^, K^+^-ATPase Activity

After electrophysiological studies, the dorsal root ganglions (L4–6) and sciatic nerves were harvested for further analysis. Crude homogenate of sciatic nerves was prepared for the measurement of Na^+^, K^+^-ATPase activity according to the coupled enzymatic method (17). The results were presented as oxidized NADH.

### Measurement of ROS, Advanced Glycation End-Products, and Antioxidant Activities

The ROS of the sciatic nerves was tested through the method described previously (19). Briefly, the homogenates of sciatic nerves were incubated with 2,7-dichlorofluorescein diacetate. Then, the absorbance of the samples was measured at 525 nm (emission wavelength) and 488 nm (excitation wavelength).

The AGE contents (20) and antioxidant activities, including glutathione-S-transferase (GST), catalase (CAT), superoxide-dismutase (SOD), and glutathione peroxidase (GPx), were measured using standard assay kits (Sigma-Aldrich, St. Louis, MO, USA)

### Hydrogen Sulfide Measurement

The H_2_S of the nerves was measured through the method described previously (19). Briefly, solutions of 0.125 ml of 1.0% zinc acetate and 0.15 ml of double-distilled water were prepared. Then, 0.067 ml of 7.2 M HCl containing 20 mM N,N-dimethyl-phenylene diamine dihydrochloride and 0.067 ml of 30 mM FeCl_3_ were added in turn. The absorbance of the samples was tested at 670 nm. The results were presented as μmol/mg protein.

### Western Blotting

The harvested dorsal root ganglions were prepared routinely. The protease inhibitors were purchased from Promega, USA. BCA assay (Thermo Scientific Inc., IL) was applied for the measurement of total protein concentration. Briefly, protein extracts were heated (100°C, 5 min) and separated by electrophoresis. After being transferred to a PVDF membrane (Bio-Rad, CA), pH 7.4 TBST buffer (100 mM NaCl, 50 mM Tris-HCl, and 0.1% Tween-20) containing 5% nonfat dry milk was added for blocking. Then, the membranes were incubated with 1:1,000 rabbit anti-rat Nrf2 antibody and 1:1,000 mouse anti-rat HO-1 in TBST buffer at 4°C overnight After being washed three times by TBST buffer (5 min each time), the membranes were incubated with 1:3,000 HRP-conjugated goat anti-rabbit or goat anti-mouse IgG for 2 h at room temperature. The primary antibodies and secondary antibodies mentioned above were purchased from Sigma, St. Louis, MO, USA. After being washed by PBS, the activity of horseradish peroxidase (HRP) was measured by an ECL kit (Millipore, USA). A Densitometer Scanner (GS 800, Bio-Rad, CA) was used to scan the image. PDQuest 7.2.0 software (Bio-Rad, CA) was used for calculating optical density. Internal control was set up using 1:10,000 Rabbit anti-rat GAPDH polyclonal antibody (Sigma- Aldrich, USA).

### Statistical Analysis

All data in this research were expressed as the mean ± standard error of mean. One-way analysis of variance (ANOVA) was applied for comparisons of the data using SPSS13.0 software (SPSS Inc., Chicago, IL, USA). Tukey *post-hoc* test was applied when comparing differences between groups. Values of *p* < 0.05 were considered as statistically significant.

## Results

### The Effect of Myricetin on Plasma Glucose

In the present study, we found that the glucose level in the diabetic control group (21.35 ± 1.66 mmol/L) was significantly higher than that in the normal control group (4.03 ± 0.39 mmol/L). After myricetin treatment that lasted for 2 weeks, the plasma glucose level decreased to 17.41 ± 0.72 mmol/L (0.5 mg/kg), 12.52 ± 0.55 mmol/L (1.0 mg/kg), and 11.36 ± 0.60 (2.0 mg/kg). The plasma glucose level of diabetic animals that received a mid dose (1.0 mg/kg) and a high dose (2.0 mg/kg) of myricetin were significantly below that in control diabetic rats (*p* < 0.05, **Supplementary Figure 3**), indicating that myricetin could reduce plasma glucose in a dose-dependent way under diabetic conditions.

### Myricetin Ameliorates Diabetic Hyperalgesia and Allodynia

**Figure 1:**
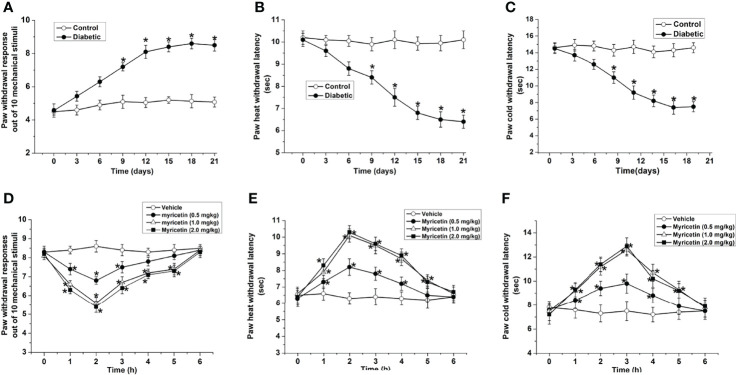
Myricetin ameliorates diabetic mechanical hyperalgesia, heat hyperalgesia, and cold allodynia (*n* = 10 for each group). The paw withdrawal response out of ten mechanical stimulations **(A)** was significantly increased, and the paw withdrawal latencies to heat **(B)** and cold stimulation **(C)** were significantly decreased by STZ treatment, indicating the development of DPN. *p < 0.05 for the comparison to normal group. Myricetin significantly decreased the paw withdrawal response out of 10 mechanical stimuli **(D)**, and increased the paw withdrawal latency to heat **(E)** and cold stimuli **(F)** in diabetic rats. *p < 0.05 for the comparison to the saline-treated diabetic rats.

STZ treatment induced obvious injury to sensory function, including hyperalgesia and allodynia to mechanical, heat, or cold stimuli (**Figures 1A–C**). Three weeks after STZ injection, the paw withdrawal responses to mechanical stimulation in STZ-treated rats were markedly higher than those in the normal control group, indicating the development of mechanical hyperalgesia in STZ-treated rats (*p* < 0.05, **Figure 1A**). Furthermore, latency to heat or cold stimuli in the STZ-treated rats was markedly lower than that in the normal control group (*p* < 0.05, **Figures 1B, C**), indicating the development of heat hyperalgesia and cold allodynia in STZ-treated rats.

Myricetin ameliorated abnormal sensation in diabetic rats. Myricetin markedly reduced the ratio of paw withdrawal responses to mechanical stimulation dose-dependently under diabetic conditions (*p* < 0.05, **Figure 1D**), indicating that myricetin could ameliorate diabetic mechanical hyperalgesia in rats. The inhibitory effect of myricetin on mechanical responses started at 1 h and ended at 5 h after myricetin administration. In addition, myricetin significantly increased the heat and cold paw withdrawal latencies (*p* < 0.05, **Figures 1E, F**), which were markedly decreased under diabetic conditions (*p* < 0.05, **Figures 1B, C**). These findings suggested that myricetin is capable of ameliorating both diabetic cold allodynia and heat hyperalgesia. The inhibitory effect of myricetin on diabetic cold allodynia and heat hyperalgesia existed from 1 h to 5 h after myricetin treatment.

### Effect of Myricetin on Nerve Morphology

**Figure 2:**
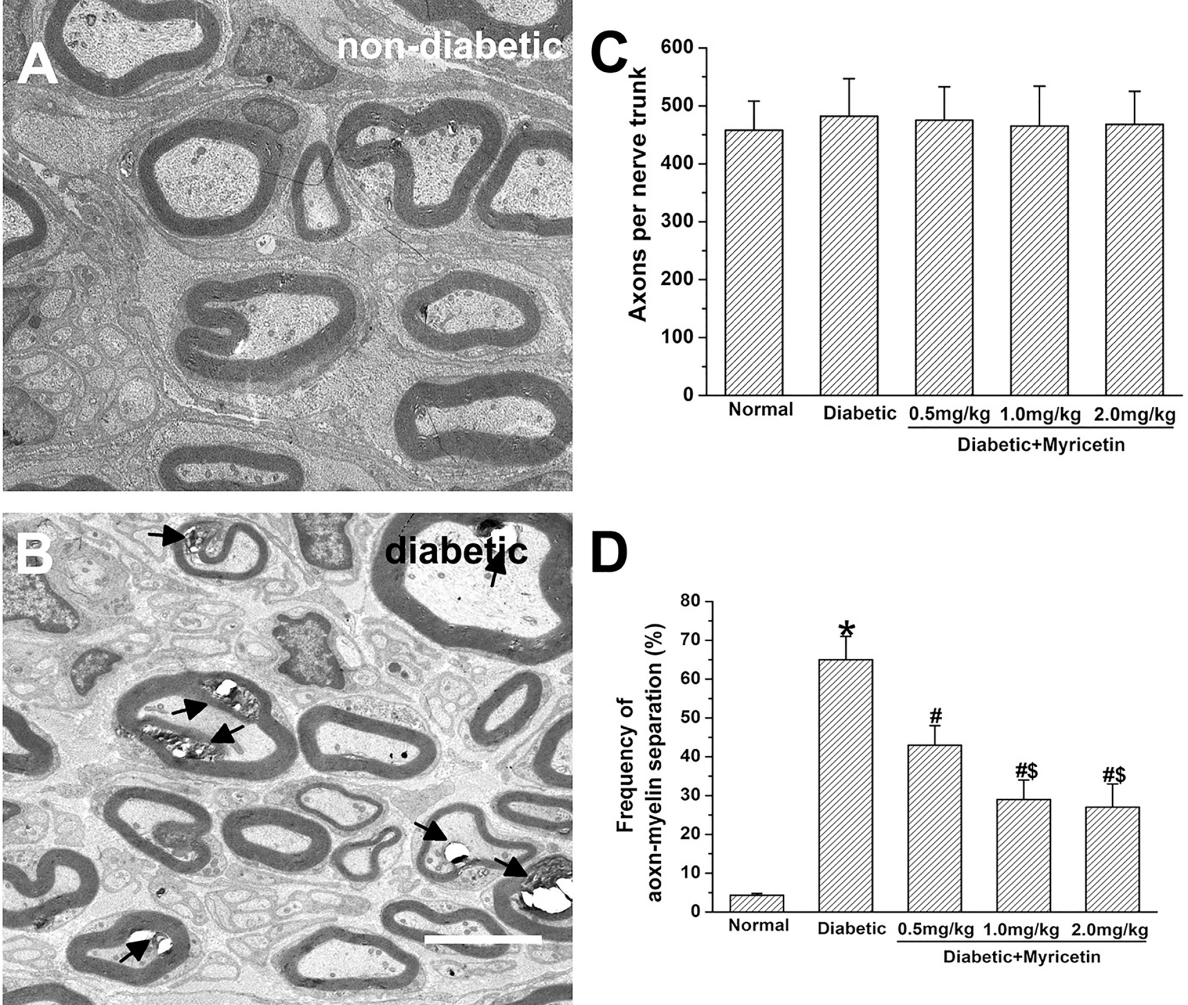
The beneficial effect of myricetin on nerve morphology in diabetic rats (*n* = 10 for each group). A significantly increased axon–myelin separation was noted in diabetic myelinated axons **(A, B)**. Myricetin at different doses has little effect on axonal number in saphenous nerve **(C)**. Application of myricetin significantly ameliorated the increased frequency of axon–myelin separations in diabetic nerves **(D)**. *p < 0.05 for the comparison to the normal group, ^#^ *p* < 0.05 for the comparison to the saline-treated diabetic rats. ^$^ *p* < 0.05 for the comparison to the diabetic rats treated with myricetin at 0.5 mg/kg.

The axonal number was in the similar range between the normal and diabetic rats. In addition, myricetin at different doses has little effect on axonal number in saphenous nerve in the present study (*p* > 0.05, **Figure 2C**). Noticeable morphological changes of nerves in diabetic rats were found under an electron microscope. A significantly increased axon-myelin separation was noted in diabetic myelinated axons (*p* > 0.05, **Figures 2A, B, D**). Application of myricetin significantly ameliorated the increased frequency of axon–myelin separations of the nerve under diabetic conditions (*p* > 0.05, **Figure 2D**). Additionally, the ratio of axon–myelin separations in myricetin groups at 1.0 mg/kg and 2.0 mg/kg was markedly lower than that in the myricetin group at 0.5 mg/kg. The results indicate that myricetin has the capability to improve altered nerve structures dose-dependently under diabetic conditions.

### Effect of Myricetin on NCV and NBF

**Figure 3:**
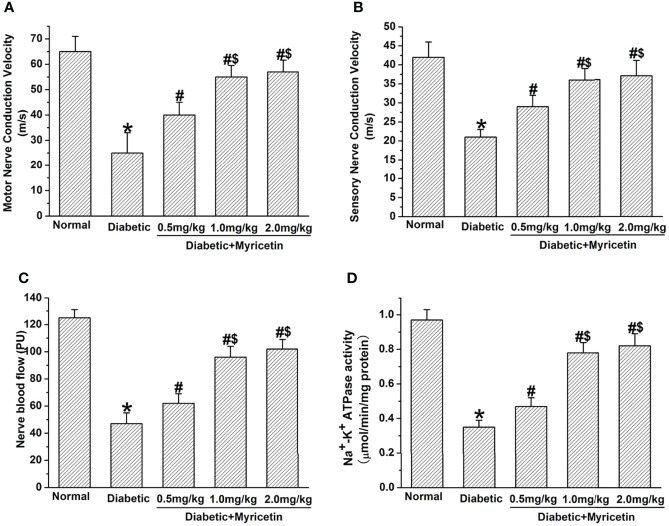
The beneficial effect of myricetin on NCV, NBF, and Na^+^, K^+^-ATPase activity in diabetic rats (*n* = 10 for each group). Myricetin significantly raised the amplitude of motor **(A)** and sensory **(B)** NVV, NBF **(C)**, and Na^+^, K^+^-ATPase activity **(D)** in diabetic rats. *p < 0.05 for the comparison to the normal group, ^#^ *p* < 0.05 for the comparison to the saline-treated diabetic rats. ^$^ *p* < 0.05 for the comparison to the diabetic rats treated with myricetin at 0.5 mg/kg.

Myricetin markedly raised NCV and NBF in diabetic rats. It was found that the sensory and motor NCV, as well as the NBF of control STZ-treated rats were significantly below those of the normal group, suggesting impaired nerve functioning under diabetic conditions (*p* < 0.05, **Figures 3A–C**). After the final administration of myricetin, the sensory and motor NCV, as well as NBF were significantly increased by myricetin at different doses (*p* < 0.05, **Figures 3A–C**). Additionally, the sensory and motor NCV, as well as NBF in the mid-dose myricetin group (1.0 mg/kg) and the high-dose myricetin group (2.0 mg/kg) were markedly better than those in the low-dose myricetin group (0.5 mg/kg). The results indicate that myricetin has the capability to improve nerve functions dose-dependently under diabetic conditions.

### Effect of Myricetin on Na^+^, K^+^-ATPase Activity

The activity of Na^+^, K^+^-ATPase significantly declined in sciatic nerves in diabetic rats at 3 weeks after STZ treatment (*p* < 0.05, **Figure 3D**). Myricetin significantly increased the declined Na^+^, K^+^-ATPase activity in sciatic nerves under diabetic conditions in a dose-dependent manner (*p* < 0.05, **Figure 3D**). The mid dose (1.0 mg/kg) and high dose (2.0 mg/kg) of myricetin attained a markedly higher activity of Na^+^, K^+^-ATPase than the low dose (0.5 mg/kg) of myricetin (*p* < 0.05, **Figure 2D**). Our findings suggest that myricetin might inhibit the oxidative damage in diabetic rats.

### Effect of Myricetin on ROS, AGEs, and Antioxidant Activity

**Figure 4:**
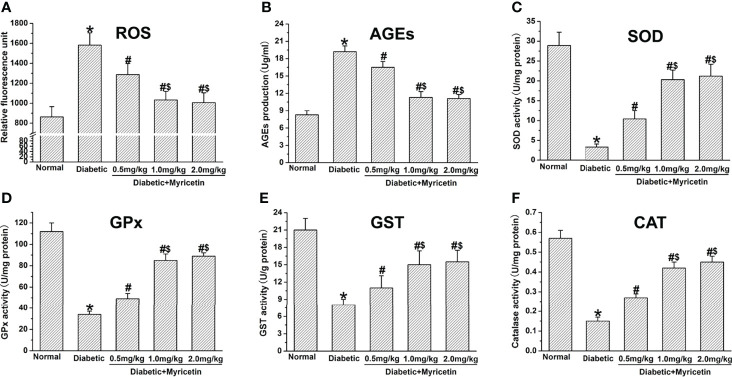
The effect of myricetin on oxidative stress in diabetic rats (*n* = 10 for each group). Myricetin significantly lowered the production of ROS **(A)** and AGEs **(B)**, and increased the activities of SOD **(C)**, GPx **(D)**, GST **(E)**, and CAT **(F)** in diabetic rats. *p < 0.05 for the comparison to the normal group, ^#^ *p* < 0.05 for the comparison to the saline-treated diabetic rats. ^$^ *p* < 0.05 for the comparison to the diabetic rats treated with myricetin at 0.5 mg/kg.

Myricetin has the capability of decreasing oxidative stress under diabetic conditions, which is a critical factor in the pathophysiology of DPN (15, 21). A significantly higher production of ROS and AGEs was found in diabetic rats compared to the normal group (*p* < 0.05, **Figures 4A, B**). Myricetin significantly inhibited the raised levels of ROS and AGEs dose-dependently in diabetic groups. The mid dose (1.0 mg/kg) and high dose (2.0 mg/kg) of myricetin achieved a significantly lower levels of ROS and AGEs than the low dose (0.5 mg/kg) of myricetin under diabetic conditions (*p* < 0.05, **Figures 4A, B**).

In this study, the effect of myricetin on antioxidant activities under diabetic conditions was further investigated. The activities of GPx, CAT, SOD, and GST were significantly inhibited in diabetic groups (*p* < 0.05, **Figures 4C–F**). Myricetin at different doses was found to raise the level of the activities of GPx, CAT, SOD, and GST dose-dependently under diabetic conditions. Myricetin at mid dose (1.0 mg/kg) and high dose (2.0 mg/kg) achieved significantly higher levels of antioxidants than that at low dose (0.5 mg/kg) (*p* < 0.05, **Figures 4C–F**). These findings suggest that myricetin has the capacity to regain the decreased antioxidant level in diabetic rats.

### Myricetin Activates H_2_S and Nfr2/HO-1 Pathway

**Figure 5:**
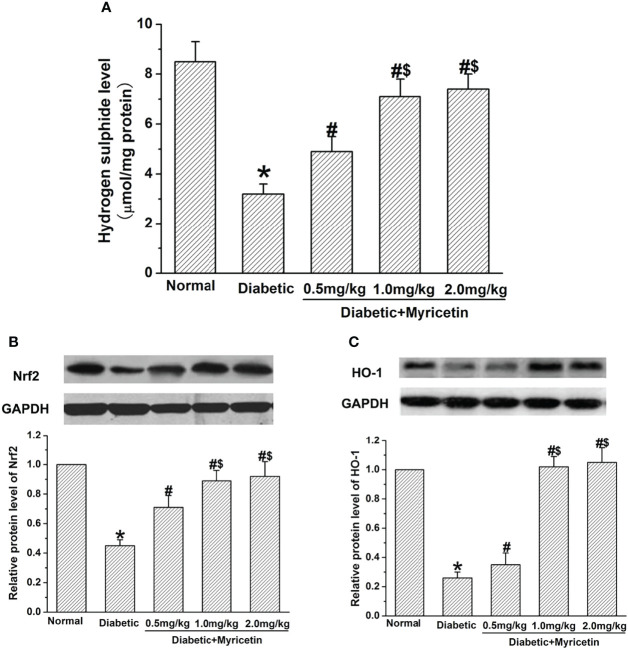
The effect of myricetin on H_2_S, Nrf2, and HO-1 under diabetic conditions (*n* = 10 for each group). STZ significantly decreased the levels of H_2_S **(A)**, Nrf2 **(B)**, and HO-1 **(C)**. Administration of myricetin significantly reduced the levels of H_2_S **(A)**, Nrf2 **(B)**, and HO-1 **(C)** under diabetic conditions. *p < 0.05 for the comparison to the normal group, ^#^ *p* < 0.05 for the comparison to the saline-treated diabetic rats. ^$^ *p* < 0.05 for the comparison to the diabetic rats treated with myricetin at 0.5 mg/kg.

Myricetin raised the level of H_2_S in a dose-dependent manner in the diabetic group (*p* < 0.05, **Figure 5A**). In diabetic rats, myricetin at mid dose (1.0 mg/kg) and high dose (2.0 mg/kg) achieved a significantly higher levels of H_2_S than that at low dose (0.5 mg/kg) (*p* < 0.05, **Figure 5A**). It has been shown that the ability of H_2_S to clear ROS and activate endogenous antioxidant enzymes is related to the Nrf2-dependent pathway. The influence of myricetin on the expression of Nrf2 and HO-1 under diabetic conditions was further investigated by Western blot. The Nrf2 expression and HO-1 expression were both inhibited under diabetic conditions, but the inhibiting effect was significantly reversed by myricetin at different doses (*p* < 0.05, **Figures 5B, C**). The mid dose (1.0 mg/kg) and high dose (2.0 mg/kg) of myricetin achieved a significantly higher level of Nrf2 expression and HO-1 expression than myricetin at low dose (0.5 mg/kg) (*p* < 0.05, **Figures 5B, C**). These results suggest that myricetin activates H2S and Nfr2/HO-1 pathway in diabetic rats.

## Discussion

The purpose of our study was to detect the potential beneficial effect of myricetin on DPN. It was proved that myricetin has the capability of attenuating diabetes-induced impairment in sensation, NCV, and NBF. In addition, myricetin significantly reduced the generation of ROS and AGEs, and raised the level of antioxidant activities in nerves, indicating that myricetin was capable of attenuating oxidative injury under diabetic conditions. Further studies showed that myricetin is able to increase H_2_S levels in nerves, as well as upregulated the Nrf2 expression and HO-1 expression in dorsal root ganglions, indicating the activation of Nrf-2-dependent endogenous antioxidant defense by myricetin. All these results suggest the potential therapeutic values of myricetin for diabetic neuropathy.

Myricetin has the capability of attenuating diabetes-induced damage in peripheral nerve function, which was evident from the improved sensation and NCV in myricetin-treated diabetic rats. In previous studies, the anti-allodynia and neural protective effects of myricetin have already been reported (22, 23). In a model of neuropathy pain in rat (spinal nerve ligation), myricetin was found to reduce the established neuropathic pain behaviors, which is related to its ability in inhibiting voltage-activated calcium channel currents (23). Neuropathic pain and oxidative neurotoxicity are two adverse main actions of DM induced by STZ (24). There are also reports that neuralgia is caused by STZ-induced inflammation. In our present study, the inhibitory effect of myricetin on diabetic cold allodynia and heat hyperalgesia existed from 1 h to 5 h after myricetin treatment. However, after myricetin treatment that lasted for 2 weeks, myricetin could reduce plasma glucose under diabetic conditions. Therefore, myricetin might relieve neuralgia by inhibiting oxidative stress and inflammation caused by STZ, but not by lowering blood glucose.

We also found that myricetin was capable of ameliorating the increased frequency of axon–myelin separations, which has been recognized as a characteristic feature of diabetic nerve (18). All these findings indicate that myricetin possesses the potential to protect neurons and nerve fibers against damage induced by hyperglycemia, which might contribute to its beneficial effect on neural functions under diabetic conditions. Furthermore, descending of NBF was also found in diabetic rats, which has been proposed to be related to impaired neural functions in DPN (25). Myricetin has been reported to be capable of inhibiting ROS-induced damage to endothelium *in vitro* (26). Thus, the potential protective effect of myricetin on vascular endothelial cells in nerve micro-vessels might contribute to its beneficial effect on NBF, which may partially explain the improvement of the electrophysiological and morphological changes by myricetin in diabetic rats.

Chronic hyperglycemia is an important initiating factor of deficit in sensory and motor NCV and other situations of DPN (27). Hyperglycemia has been found to result in a series of cellular and tissue damage in nerve tissue, which lead to the development of DPN. In our present study, myricetin has the capability of decreasing plasma glucose under diabetic conditions, which is consistent with past research (28, 29). The lowered plasma glucose in myricetin-treated rats was expected to result in less damage to nerve cells and tissues, which might partly explain the beneficial effect of myricetin on DPN.

Oxidative stress is a critical factor in diabetes and DPN (3). Excess generation of ROS has been thought to be the reason for the abnormal morphological change of axons, alteration of neural permeability, and functional modification of multiple cellular proteins (4). In consideration of the potent antioxidative activity of myricetin, we were interested in the relationship between the inhibiting effects of myricetin on oxidative stress and its beneficial effects on neural function under diabetic conditions. We found that myricetin significantly decreased the production of ROS and AGEs under diabetic conditions, suggesting that myricetin has the capability of decreasing oxidative stress status in nerves in diabetic rats. In addition, myricetin was found to raise the activity level of antioxidants (SOD, GST, GPx, and CAT), indicating that myricetin is capable of recovering the antioxidative activity under diabetic conditions. All these results indicate that the antioxidative effect might be the underlying mechanism of the beneficial effect of myricetin on impaired nerve functions. Furthermore, myricetin significantly raised the activity of Na^+^, K^+^-ATPase in the sciatic nerve under diabetic conditions. As Na^+^, K^+^-ATPase plays an important role in cell homeostasis (17), it is important to restore its normal activity (30). The oxidative damage to Na^+^, K^+^-ATPase activity has been proved to be a key point in research (1). Thus, it is likely that myricetin might also inhibit the oxidative damage to Na^+^, K^+^-ATPase, just as the antioxidative effect of myricetin on nerves in this study. Future research should be done to confirm such a speculation.

The mechanism underlying the finding that myricetin is capable of activating endogenous antioxidant defenses in diabetic rats has been unclear thus far. It has been proved that the Nrf2 pathway plays a very important role in the defense system of the organism (5, 8). Recently, Nrf2 has gained more and more attention as an important target in diabetes and its complications (6, 9). The protective effect of Nrf2 has been proved in β cells of pancreas (7), kidney (8), and cardiomyocytes (9) against oxidative damage under high-glucose conditions. Previous studies have found that myricetin significantly protected the mouse heart from lipopolysaccharide (LPS)-induced injuries *in vivo* and *in vitro*, and the underlying mechanisms have been demonstrated to be associated with the inhibition of IκBα/NF-κB activity and attenuation of inflammatory cytokine secretion, such as IL-1α, IL-1β, TNF-α, and MCP-1. Up to now, lines of evidence implicating the role of Nrf2 in diabetic neuropathy have been relatively lacking. A previous study and the present study have found decreased Nrf2 expression in diabetic nerves (6), suggesting inhibition of the Nrf2 pathway in diabetic nerves. In addition, the results of the present study indicated that Nrf2 expression in nerves was upregulated by myricetin under diabetic conditions, which could activate the antioxidant defense system (SOD, GST, GPx, CAT, and a phase II detoxifying enzyme HO-1) to attenuate reactive radical-induced damage. However, the underlying mechanisms in it need to be further studied. Furthermore, H_2_S has been proved to be capable of directly eliminating ROS and activating an antioxidant system in the Nrf2 pathway (31). Further examination of the effect of myricetin on H_2_S showed that myricetin is capable of increasing the level of H_2_S in diabetic nerves, indicating the participation of H_2_S in the beneficial effect of myricetin on DPN through an Nrf2-dependent manner. Therefore, it is proposed that myricetin might be effective as a plant-based anti-hyperglycemic and neuroprotective agent and it deserves more pharmacological exploration in future.

## Conclusions

Myricetin could restore the impaired sensory and motor functions under diabetic conditions. The Nrf2-dependent antioxidant action and the capability of decreasing plasma glucose might be the underlying mechanisms for the beneficial effect of myricetin on impaired neural functions. All the results suggest the potential therapeutic value of myricetin for DPN. Nevertheless, the beneficial effect of myricetin on DPN should be confirmed by more evidence in larger-animal models or even humans before clinical application.

## Data Availability Statement

The original contributions presented in the study are included in the article/**Supplementary Material**. Further inquiries can be directed to the corresponding authors.

## Ethics Statement

The animal study was reviewed and approved by the Institutional Ethical Committee of General Hospital of Northern Theater Command.

## Author Contributions

HY and LX contributed to the study conception and design. Material preparation, data collection, and analysis were performed by JM and YC. The first draft of the manuscript was written by JM and all authors commented on previous versions of the manuscript. The manuscript revision was conducted by JM. The funding source and study supervisor were from LX. All authors have read and agreed to the published version of the manuscript.

## Funding

This research was supported by the Natural Science Foundation of Liaoning Province (2019-ZD-1028).

## Conflict of Interest

The authors declare that the research was conducted in the absence of any commercial or financial relationships that could be construed as a potential conflict of interest.

## Publisher’s Note

All claims expressed in this article are solely those of the authors and do not necessarily represent those of their affiliated organizations, or those of the publisher, the editors and the reviewers. Any product that may be evaluated in this article, or claim that may be made by its manufacturer, is not guaranteed or endorsed by the publisher.
