# Supplementary material for: Myricetin Improves Impaired Nerve Functions in Experimental Diabetic Rats
Source: Front Endocrinol (Lausanne). 2022 Jul 19;13:915603. doi: 10.3389/fendo.2022.915603 (PMC9343592; doi:10.3389/fendo.2022.915603)
Supplement: Supplementary file 1 [file DataSheet_1.docx]

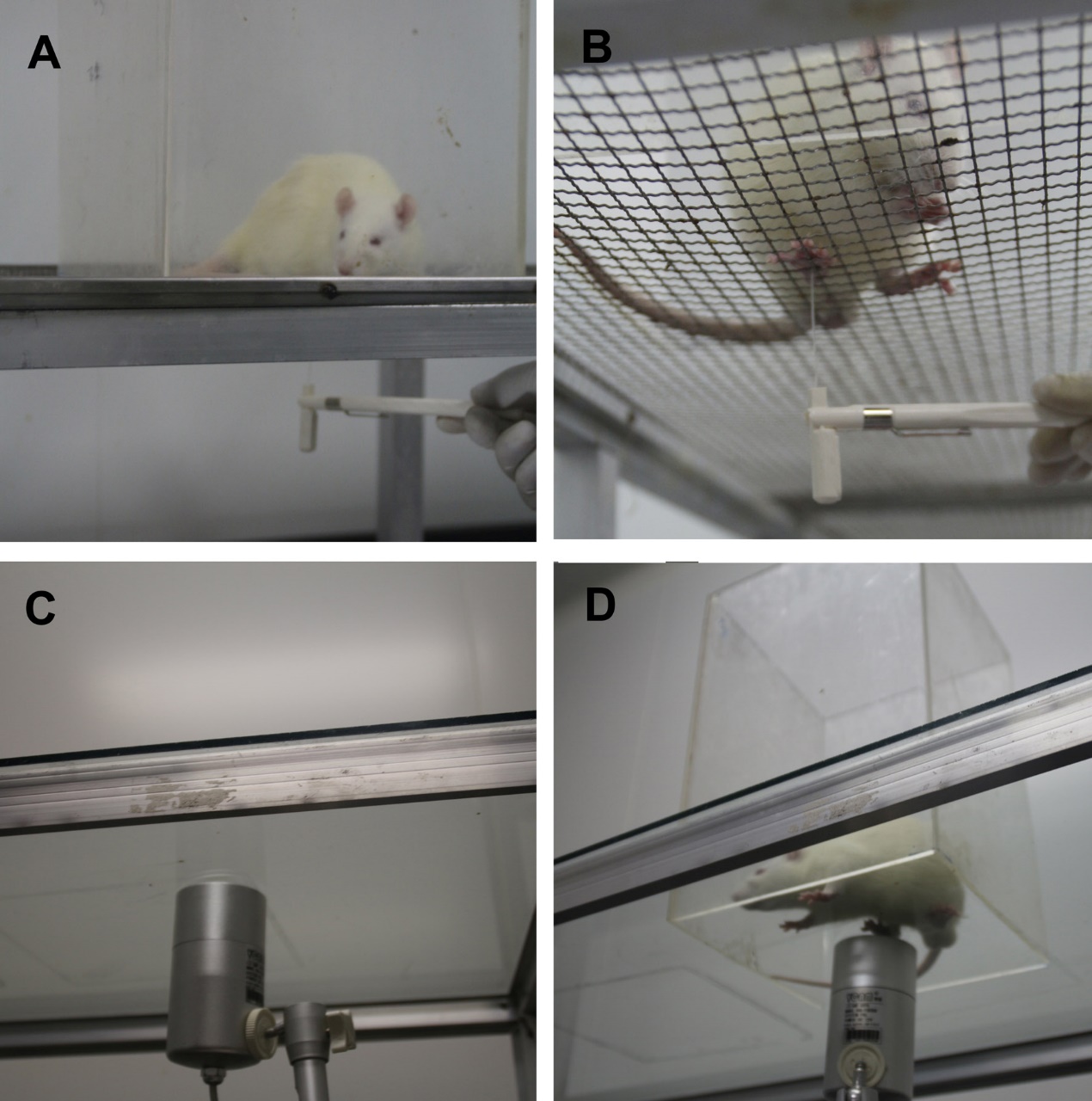


Supplemental Fig. 1. Evaluation of Mechanical nociception by von Frey filament (A, B) and Heat nociception by hot plate test (C, D).


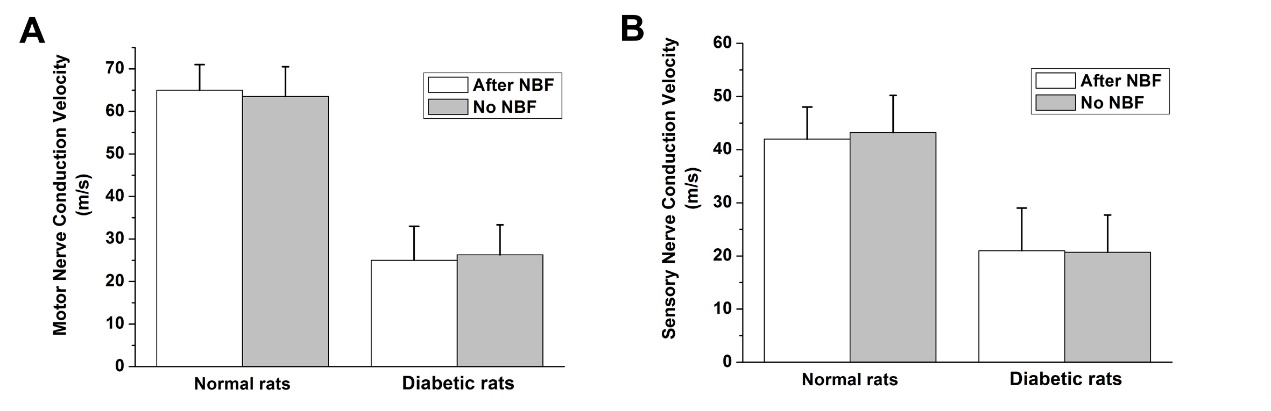


Supplemental Fig. 2. NBF measurement has little effect of MNCV (A) and SNCV (B) in both normal and diabetic rats.


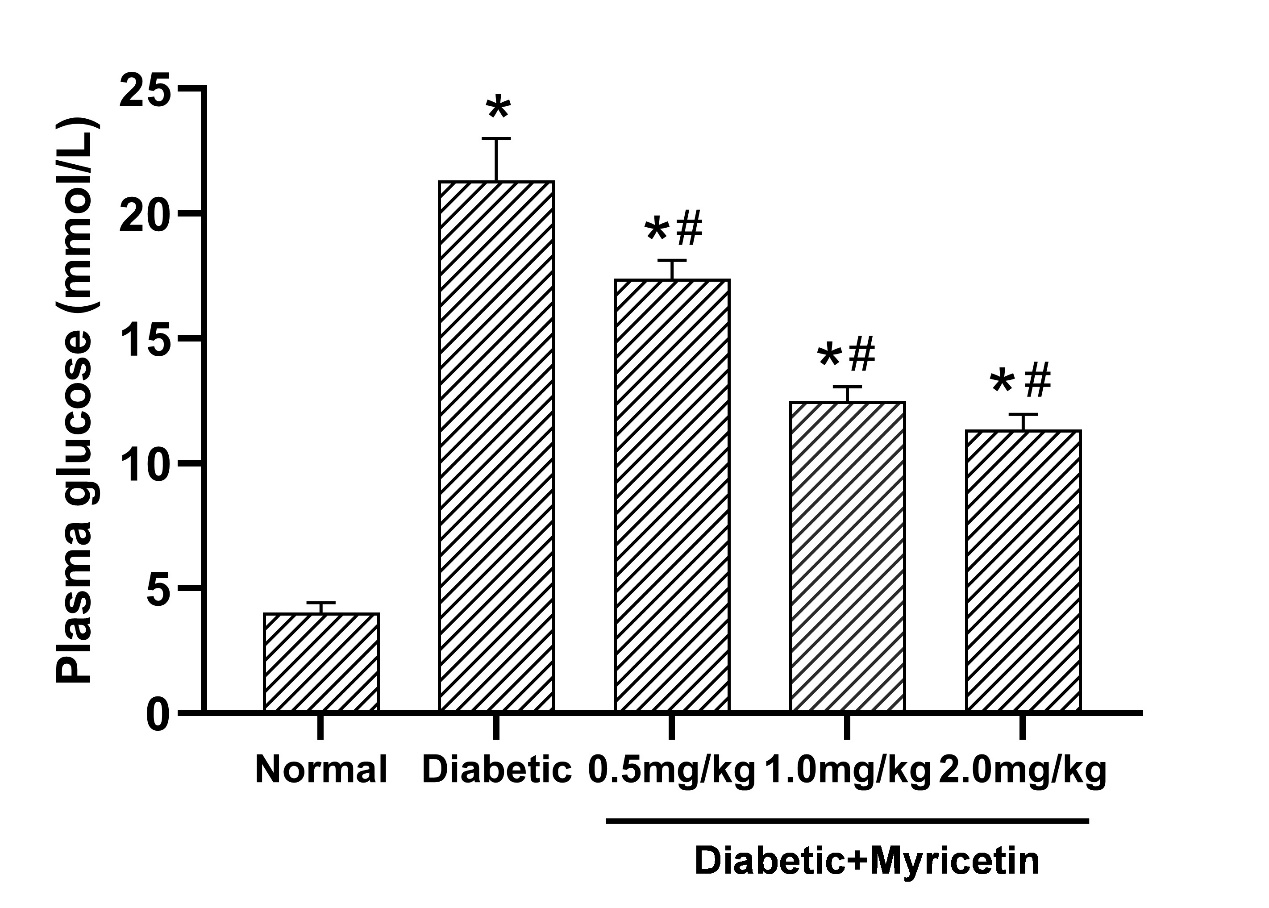


Supplemental Fig. 3. The effect of myricetin on plasma glucose under diabetic condition.
